# Case report: A case of corneal deposits between binocular descemet membrane and corneal endothelial layer after small-incision lenticule extraction (SMILE) followed by HPV vaccine

**DOI:** 10.3389/fmed.2022.1042405

**Published:** 2022-12-21

**Authors:** Hao Zhang, Yingping Deng, Ke Ma, Chengshu Sun, Jing Tang

**Affiliations:** Department of Ophthalmology, West China Hospital, Sichuan University, Chengdu, Sichuan, China

**Keywords:** small-incision lenticule extraction, corneal deposit, gene mutation, human papillomavirus vaccine, congenital corneal dystrophy

## Abstract

**Background:**

Deposits located between the Descemet memberane and the corneal endothelial layer in both eyes has not yet been reported after SMILE.

**Case report:**

Grayish white fine deposits was found between the Descemet memberane and the corneal endothelial layer in both eyes of the patient's cornea, and no other abnormalities were observed in the anterior and posterior segments of patient's eyes with ophthalmic examination instrument. However, the visual acuity of patient remained unchanged. Significantly, the patient had a sister who had undergone the same procedure a year earlier without any complications. After careful questioning, we learned that the patient received the 9-valent human papillomavirus (HPV) vaccine on the third post-operative day without any other special experiences. We performed immunological examination and genetic testing on the patient. The results of immunological examination of patient showed no obvious abnormality, which was consistent with the routine trend after vaccination. In particular, a homozygous variation of the ARSG gene was found in the patient and her sister.

**Conclusion:**

There are two possible causes of corneal changes in patients. The first is IGA elevation caused by vaccination, deposited in the cornea. Second, the ARSG gene mutation of the patient leads to a potential congenital corneal dystrophy, and clinical manifestations occur under the stimulation of the vaccine.

## Background

Small-incision lenticule extraction (SMILE) is a well described laser refractive surgery that was widely used in treating myopia since its first application in 2011 (1). In short, a femtosecond (FS) laser creates an intrastromal lenticule, which is extracted through a corneal incision. Although post-operative haze, prominent corneal ectasia, diffuse lamellar keratitis, and stromal keratitis have been documented (2), deposits located between the Descemet memberane and the corneal endothelial layer in both eyes has not yet been reported after SMILE.

## Case presentation

A 26-year-old woman had small-incision lenticule extraction in both eyes on January 26, 2022. The preoperative uncorrected distance visual acuity (UDVA) was 20/333 and 20/250 in the right eye and left eye, the preoperative refraction was −4.75 sphere and −4.00 sphere, respectively, and the corrected distance visual acuity (CDVA) was 20/16 in both eyes. Slit-lamp and dilated fundus examinations were unremarkable, with no signs of dryness or superficial punctate keratopathy. Intraocular pressure (IOP) was 16.6 mmHg OD and 15.3 mmHg OS. The cornea thickness was 504 mm in the right eye and 499 mm in the left eye. The SMILE procedure was done using the Visumax femtosecond laser (Carl Zeiss Meditec AG). The cap diameter was 8.0 mm, with an intended thickness of 97 μm OD and 92 μm OS, and the optical zone in both eyes was 6.5 mm. No intraoperative complications were reported. 0.5% Levofloxacin drops (Santen pharmaceutical Co, Japan) were used for the first 7 days and 0.1% Tobramycin dexamethasone, Alcon, Belgium) were used for the first 7 days, 4 times a day, then tapering the dose during the next 3 weeks.

**Figure 1:**
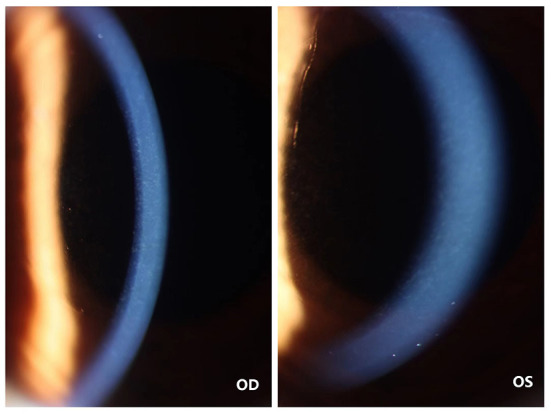
Photographs of the cornea of the patient's both eyes. The color cornea photograph the patient's both eyes showing grayish white fine deposits between the Descemet memberane and the corneal endothelial layer.

**Figure 2:**
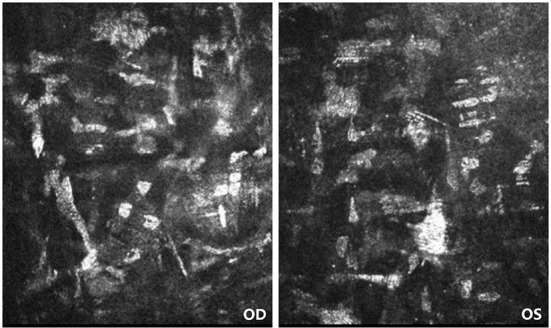
Confocal microscopy of patient's both eyes. Confocal microscopy showing the presence of hyper-reflective deposits that appeared as irregular snowflake-like images between the Descemet memberane and the corneal endothelial layer.

**Figure 3:**
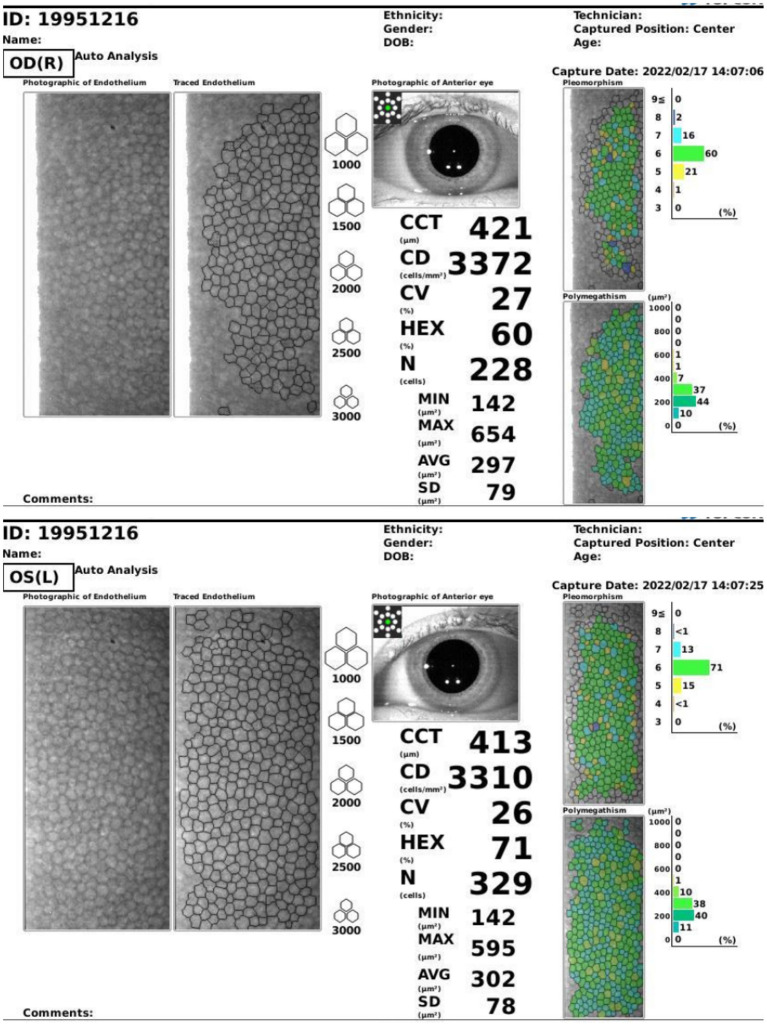
Specular microscope of both eyes of the patient. The morphology and quantity of corneal endothelium in both eyes showed no abnormality.

On the first post-operative day, the patient's cornea was in good condition without any complications. The patient underwent the second reexamination in our department 15 days after surgery. What we saw under the slit lamp was grayish white fine dust similar to keratic precipitates (KP) (Figure 1). At first, we all thought it was KP, but there was no aqueous flare, inflammation, corneal edema or interface debris. No other abnormalities were observed in the anterior and posterior segments of patient's eyes with ophthalmic examination instrument. Moreover, her visual acuity remained unchanged. She was prescribed a week-long tapering dose of Tobramycin and Dexamethasone Eye Drops (s.a. Alcon-Couvreur n.v.) beginning at qid for the first week. At 1-week follow-up, her ocular examination showed no obvious change, and there were still a large number of scattered blotchy deposits of material without improvement, but her reported no loss of vision or any discomfort. It's worth noting that the patient had a sister who had undergone the same procedure a year earlier without any complications. After careful questioning, we learned that the patient received the 9-valent human papillomavirus (HPV) vaccine on the third post-operative day without any other special experiences. Confocal microscopy performed for both eyes revealed the presence of hyper-reflective deposits that appeared as irregular snowflake-like images between the Descemet memberane and the corneal endothelial layer (Figure 2), with normal endothelial morphology and quantity (Figure 3).

Absolute count examination of T cell, B cell and NK cell, quantitative detection of immunoglobulin, complement, prolactin factor B (PFB) and C-reactive protein (CRP), and detection of extractable nuclear antigen (ENA) and antinuclear antibody (ANA) were performed on patient. The results showed no obvious abnormality, which was consistent with the routine trend of immunological examination after vaccination.

We also performed trio-Whole-exome sequencing (WES) on the patient. A homozygous variation of [c. 280 (exon3) G > A (NM_001267727)] of the ARSG gene was found in the WES data of the patient. According to the ACMG mutation rating guidelines, the variation is rated as uncertain significance (VUS), and it was predicted to be harmful by a number of software. So is her sister.

The patient was reexamined in our department about 6 months after surgery. However, her ocular examination showed no obvious change compared with the previous one, and there were still a large number of scattered blotchy deposits of material without significant reduction.

## Discussion and conclusion

We report a case of corneal deposits between binocular Descemet membrane and corneal endothelial layer after small-incision lenticule extraction followed by HPV vaccine, which is the first report of this type of corneal morphological change, distinguished from several corneal lesions described later. According to the existing literature, several reasons for corneal changes similar to those in our patient are as follows.

## Immune related factors

Paraproteinemic crystalline keratopathy, a large class of diseases similar to this patient's corneal changes, is a term used to describe corneal involvement with deposits in lymphoproliferative disorders associated with hyper-gammaglobulinemia (3–6). On slit-lamp examination the corneal deposits could be found at any level from the epithelium to the deep stromal layers taking the form of tiny dots (3–5) or as a lattice-like pattern (7). The deposits could be of mid-peripheral location sparing the center. The origin of these crystalloid deposits was suggested to be hematogenous, through molecular diffusion from the limbal vascular loops (8), and they are mostly composed of IgG kappa light chain (6). All previously reported cases of paraprotein crystalline keratopathy were seen in systemic disorders associated with hypergammaglobulinemea, hence resulting in bilateral involvement. Alomar, TS had also reported a case of localized bilateral conjunctival B cell lymphoma with corneal crystalline deposits not associated with paraproteinemia (9). As mentioned above, abnormally elevated IGG can lead to corneal crystal deposition. The amount of IGA in this patient was significantly elevated, which could cause the appearance of corneal deposits through a similar mechanism. Anti-Ro-52 antibody is a common autoantibody in the ANA antibody spectrum and may be positive in normal patients or in patients with autoimmune diseases. Only the anti-Ro-52 antibody is positive, while other autoantibodies are negative, and the patient has no corresponding clinical manifestations, then the positive antibody has no special clinical significance in this case.

## Congenital corneal dystrophy

There are currently two kinds of reported diseases of congenital corneal dystrophy similar to the corneal changes in our case.

(1) Schnyder corneal dystrophy disease (SCD). The clinical characteristics of SCD are the deposition of cholesterol and phospholipids in the subepithelium and in the stroma of the cornea, resulting in corneal opacity (10–13). The specific clinical manifestations are as follows: (1) It is an autosomal dominant genetic disease with a high degree of extrinsic dominance, so there is often a family history (14). (2) The disease usually starts at about 20 years old, and a few can be 10 years younger (15). (3) Often binocular disease, the degree of corneal turbidity increases with age, so the vision is progressive decline, there may be glare and photophobia (16). (4) Limbal lipid opacity resembles cornea arcus senilis (16). (5) About 54% of SCD patients had crystalline deposition of cholesterol in the cornea (16). (6) About 4% of SCD patients have genu valgus, spinal and finger malformations and other signs. (7) About 66% of SCD patients had dyslipidemia (16–19). (8) The pathogenesis of SCD may be related to local lipid metabolism defects caused by UBIADI gene mutation, but the exact mechanism is not clear (11, 13, 14).
(2) Pre-descemet corneal dystrophy (PDCD) is a rare form of stromal dystrophy of the cornea characterized by dense, irregular deposits of opaque material between the deep stromal layer and the descemet membrane and associated with mutations in the STS gene (20). PDCD has several subgroups, which may represent sporadic, age-related, or degenerative changes (21). The symptoms of PDCD patients are mild, and their vision is generally not affected (22).

WES clearly revealed that the patient only had mutation in ARSG gene, which were inconsistent with the above two mutant genes of congenital corneal dystrophy, so we ruled out the possibility that the patient had the above two diseases. The pathogenic variation of ARSG gene can lead to type IV Usher syndrome (USH), which is an atypical form of USH. It is characterized by delayed retinitis pigmentosa (RP), night blindness, peripheral vision loss, spicule pigmentation, pigment mass, retinal annular atrophy, delayed and progressive sensorineural hearing loss (SNHL), with or without vestibular dysfunction (23–27). Although corneal abnormalities had not been reported to be associated with homozygous variation of ARSG gene, since the homozygous variation of our genetic locus is different from all previously reported loci, it can't be ruled out as one of the atypical manifestations of this gene mutation. The patient maight have congenital corneal dystrophy preoperatively, just without corresponding clinical manifestation. After being disturbed by surgery, vaccine and other factors, the clinical manifestation appears.

## Use of certain drugs, such as hydroxychloroquine

Medication with members of the chloroquine family (chloroquine, hydroxychlorique, amodiaquine) may produce ocular toxicity involving the cornea (vortex keratopathy), and corneal deposits (verticillata) can be demonstrated in most patients taking chloroquine, but these changes very rarely impair vision (28). The golden-brown deposit had been found in the hydroxychloroquine-laden cornea. Hyperreflective abnormal particles were found in different layers of the cornea by confocal microscopy and the deposits were present within the superficial epithelium, basal epithelium and anterior stroma (29). A similar distribution was noted in chloroquine (30) and amiodarone-induced keratopathy (31). Since our patient had no history of use of special drugs post-operatively, we first excluded the influence of drugs.

In conclusion, we believe that there are two possible causes of corneal changes in patients. The first is IGA elevation caused by vaccination, deposited in the cornea. Second, the ARSG gene mutation of the patient leads to a potential congenital corneal dystrophy, and clinical manifestations occur under the stimulation of the vaccine. We report this case in the hope that doctors who have encountered similar cases in the past or in the future will be able to further verify our speculation, so as to improve the perioperative management of patients who want to undergo corneal refractive surgery.

## Data availability statement

The original contributions presented in the study are included in the article/Supplementary material, further inquiries can be directed to the corresponding author/s.

## Ethics statement

The studies involving human participants were reviewed and approved by Biomedical Ethics Sub-Committee of West China Hospital of Sichuan University. The patients/participants provided their written informed consent to participate in this study. Written informed consent was obtained from the individual(s) for the publication of any potentially identifiable images or data included in this article.

## Author contributions

JT interpreted the patient data, provided the patient material, and interpreted the clinical and imaging data. HZ wrote the first draft of the manuscript. YD supplemented, perfected, and revised the first draft of the manuscript. KM and CS made a contribution to the follow-up of the patient. All authors read and approved the final manuscript.
