# Supplementary material for: Case report: A case of corneal deposits between binocular descemet membrane and corneal endothelial layer after small-incision lenticule extraction (SMILE) followed by HPV vaccine
Source: Front Med (Lausanne). 2022 Dec 21;9:1042405. doi: 10.3389/fmed.2022.1042405 (PMC9811408; doi:10.3389/fmed.2022.1042405)
Supplement: Supplementary file 1 [file Data_Sheet_1.pdf]

20220218ICU_11-1ICU

Protocol Naive Memory20220414 - Plot Sheet 1

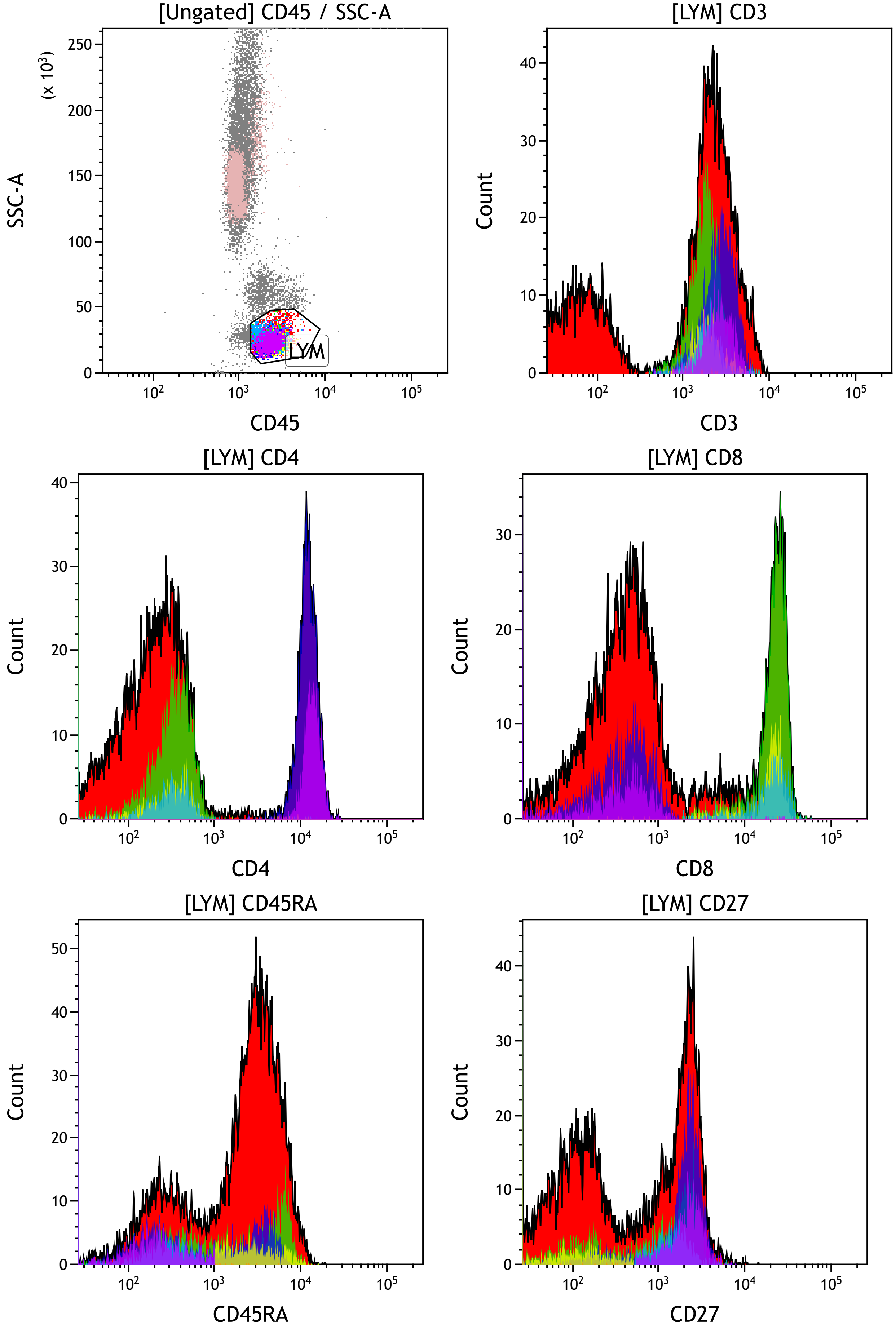

20220218ICU_11-1ICU
Protocol Naive Memory20220414 - Plot Sheet 1

[Ungated] CD4 / CD8

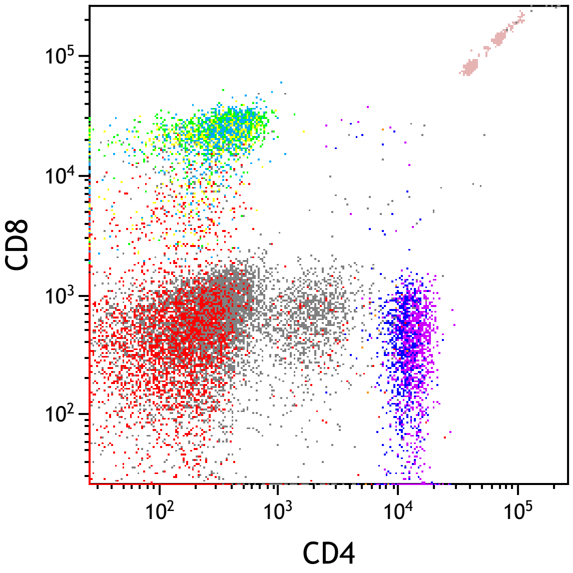

[LYM] CD4 / SSC-A

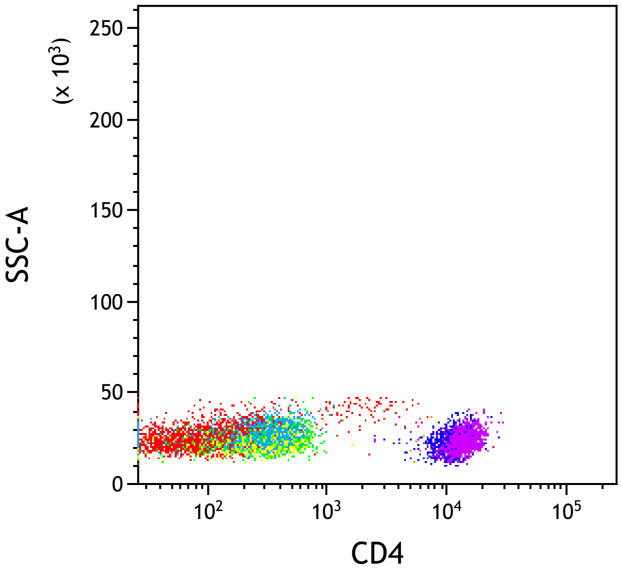

[LYM] CD8 / SSC-A

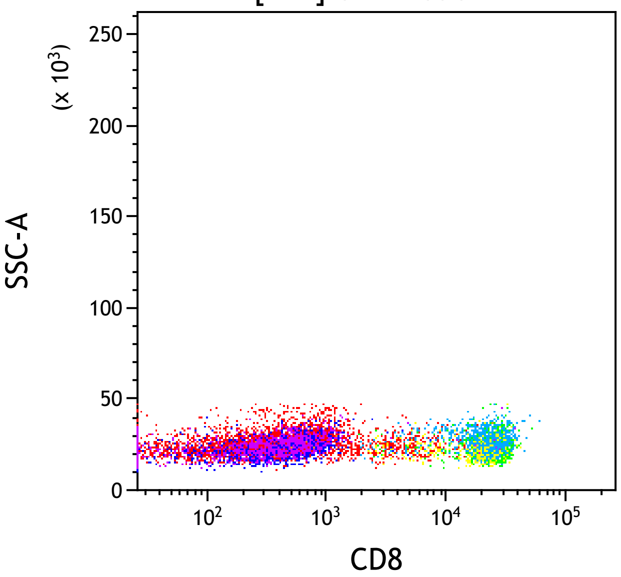

[LYM] CD3 / CD4

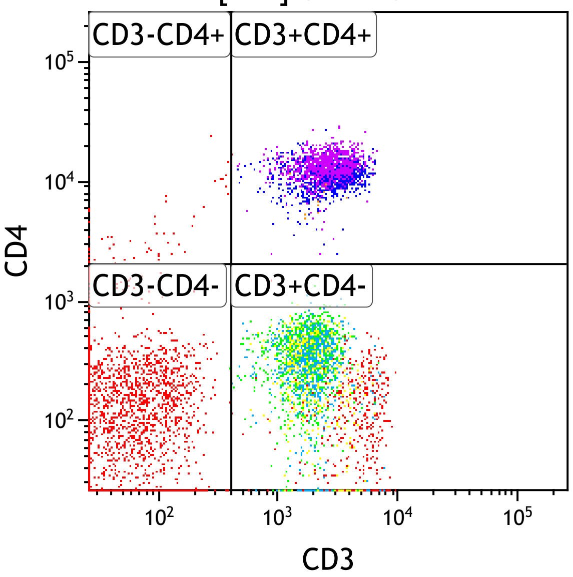

[LYM] CD3 / CD8

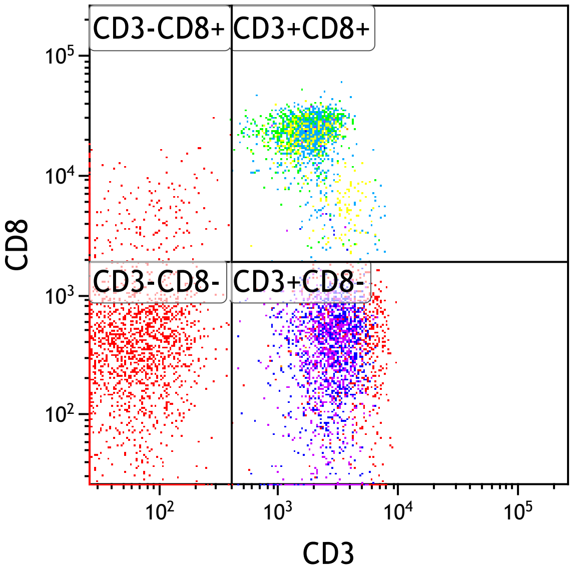

[CD3+CD4+] CD45RA / CD27

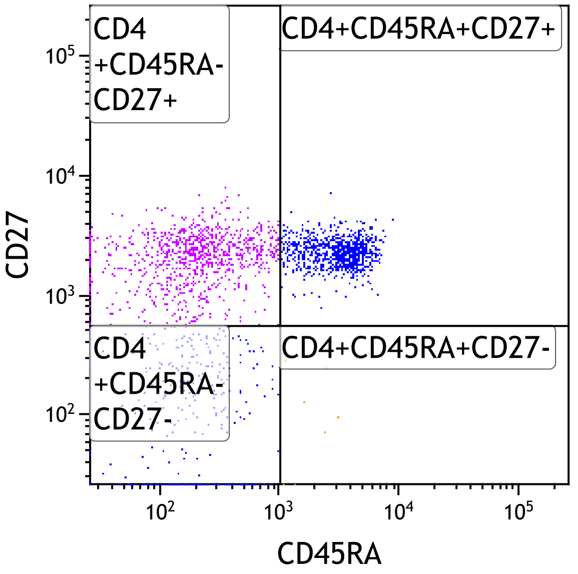

20220218ICU_11-1ICU
Protocol Naive Memory20220414 - Plot Sheet 1

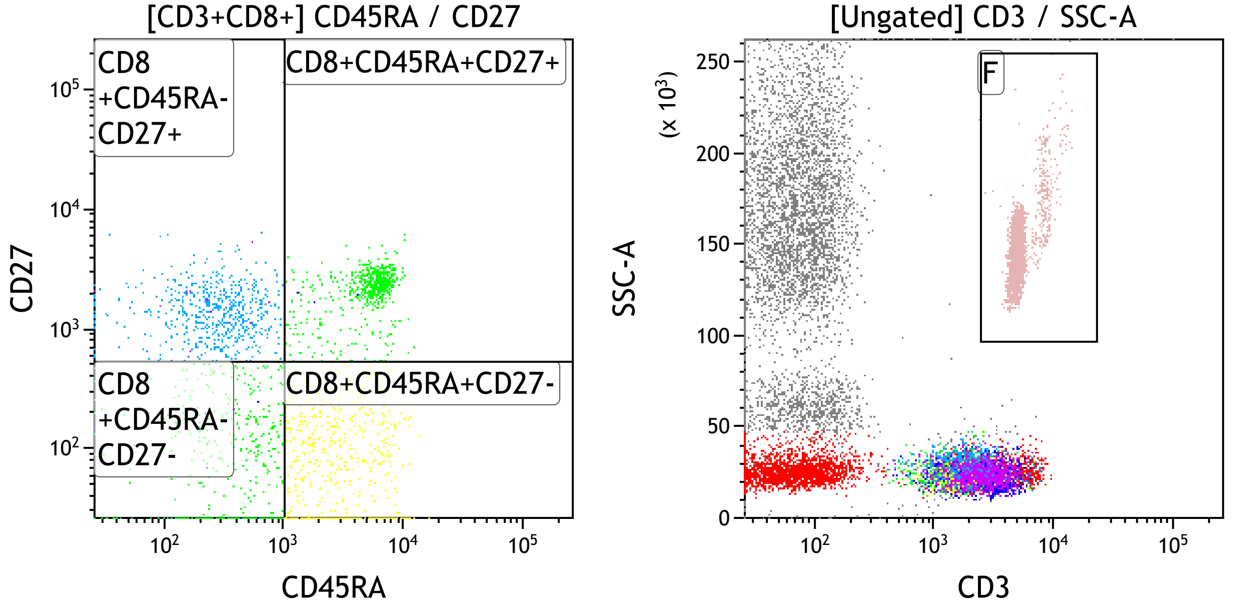

Gate	Number	%Total	%Gated	Logic
All	21,451	100.00	100.00	Ungated
F	3,587	16.72	16.72	F
LYM	9,408	43.86	43.86	LYM
CD3+CD4-	2,847	13.27	30.26	CD3+CD4- AND LYM
CD3+CD4+	2,121	9.89	22.54	CD3+CD4+ AND LYM
CD4+CD45RA+CD27-	11	0.05	0.52	CD4+CD45RA+CD27- AND CD3+CD4+ AND LYM
CD4+CD45RA+CD27+	870	4.06	41.02	CD4+CD45RA+CD27+ AND CD3+CD4+ AND LYM
CD4+CD45RA-CD27-	304	1.42	14.33	CD4+CD45RA-CD27- AND CD3+CD4+ AND LYM
CD4+CD45RA-CD27+	936	4.36	44.13	CD4+CD45RA-CD27+ AND CD3+CD4+ AND LYM
CD3+CD8-	2,694	12.56	28.64	CD3+CD8- AND LYM

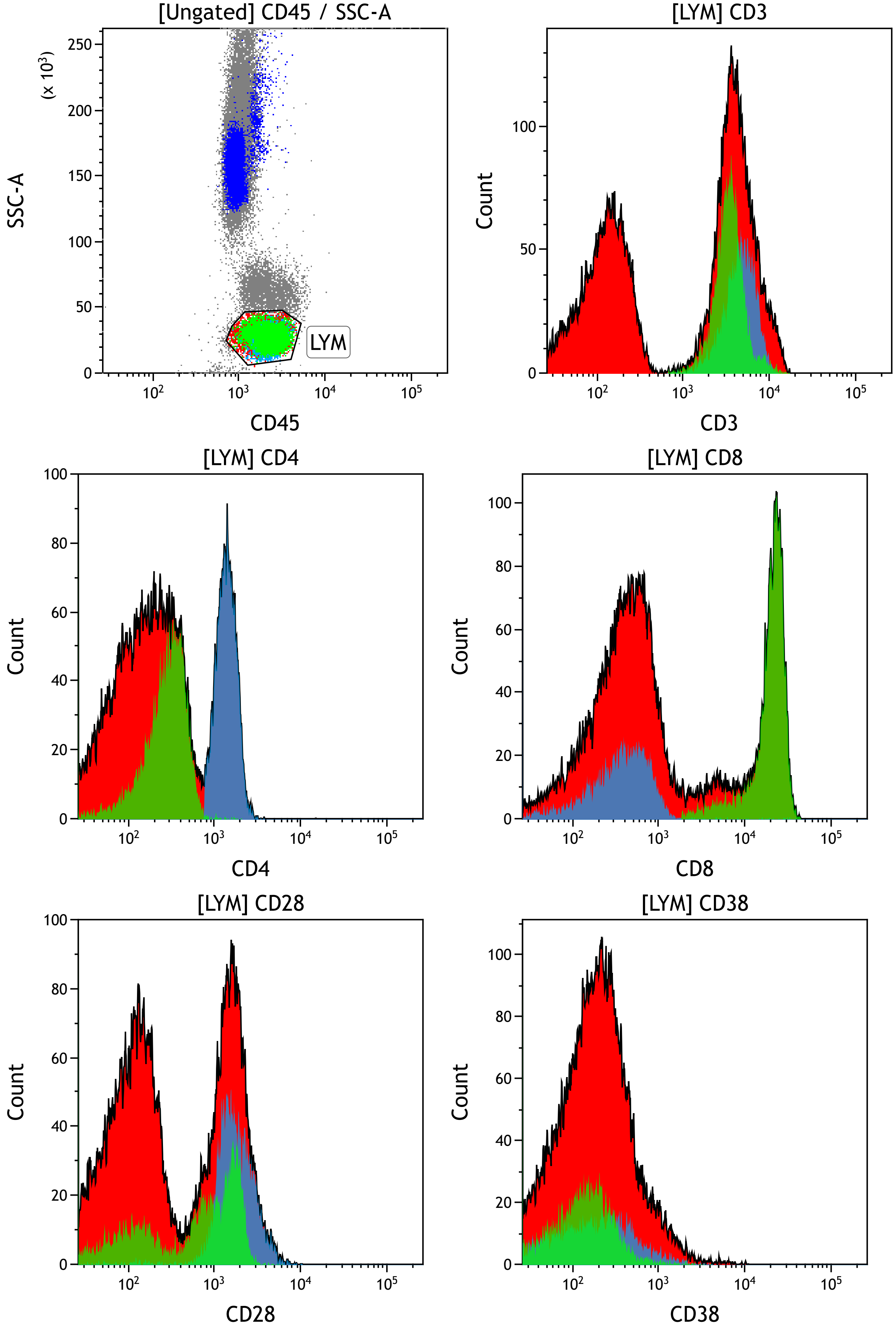
[Ungated] CD45 / SSC-A
(x 10³)
SSC-A
CD45
LYM
[LYM] CD3
Count
CD3
[LYM] CD4
Count
CD4
[LYM] CD8
Count
CD8
[LYM] CD28
Count
CD28
[LYM] CD38
Count
CD38

[LYM] HLA-DR

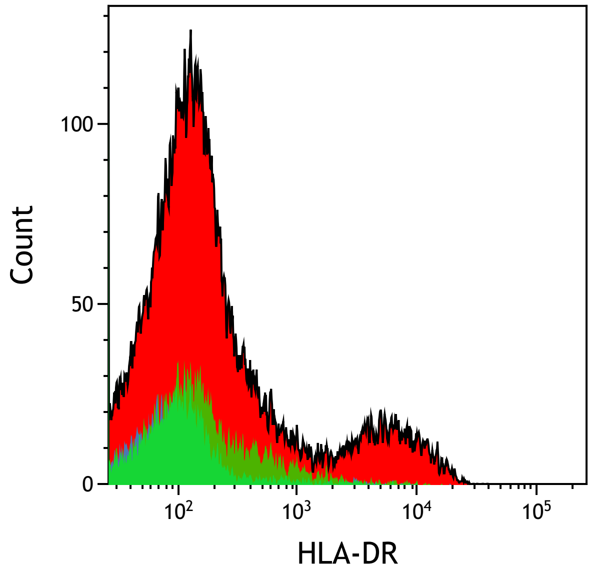

[Ungated] CD3 / SSC-A

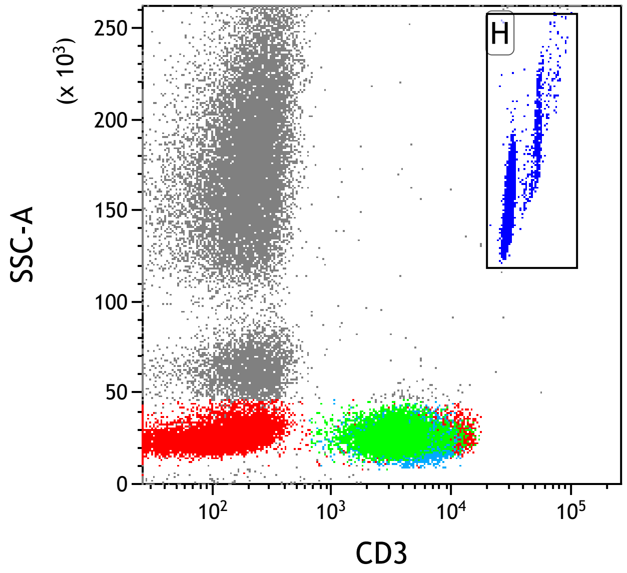

[Ungated] FSC-A / SSC-A

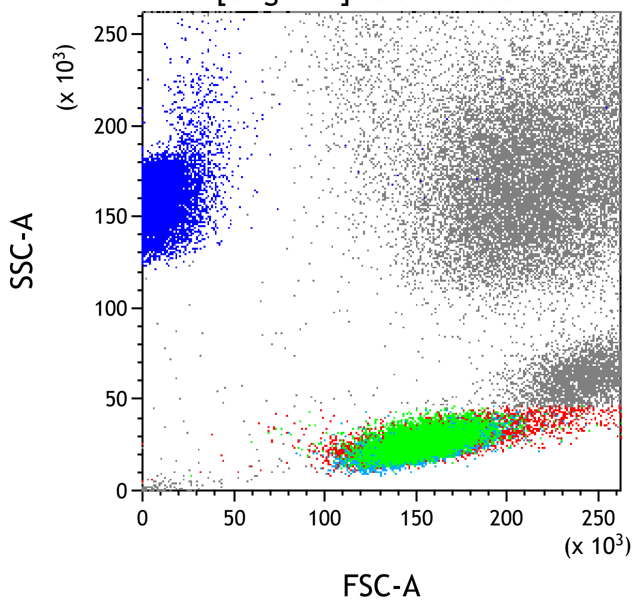

[LYM] CD3 / CD4

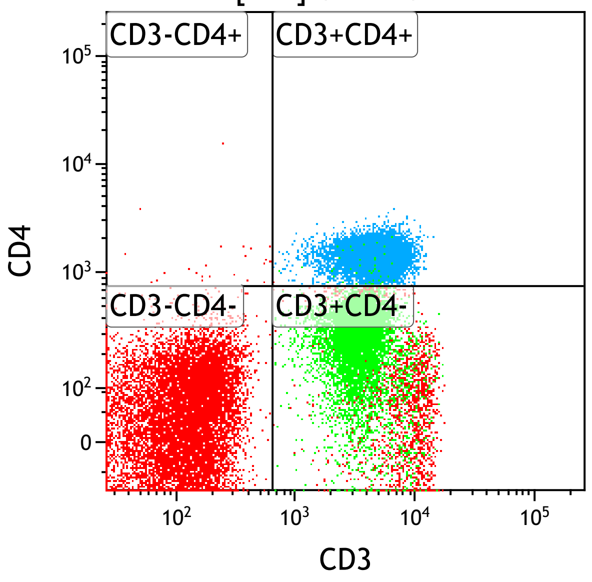

[LYM] CD3 / CD8

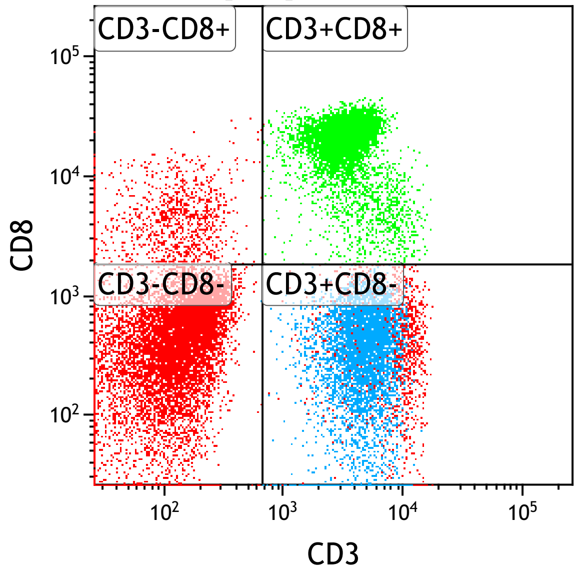

[CD3+CD4+] CD4 / CD28

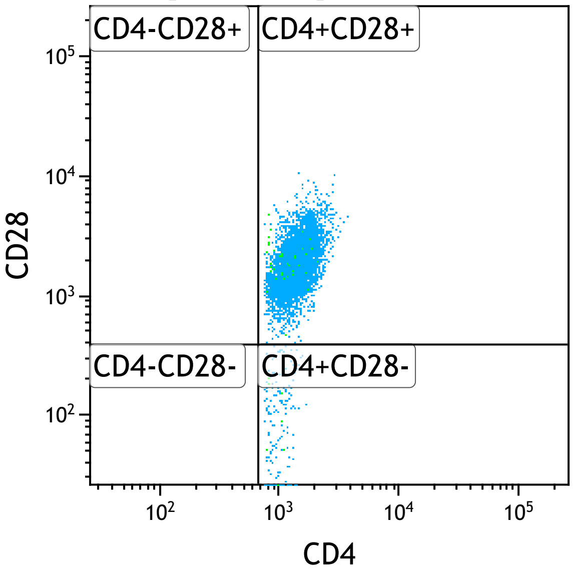

20220218ICU_11-1ICU
Protocol Active - Plot Sheet 1

[CD3+CD4+] CD4 / CD38

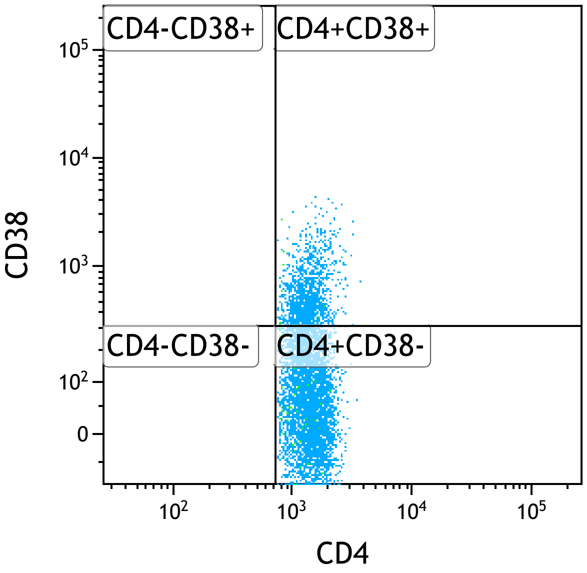

[CD3+CD4+] CD4 / HLA-DR

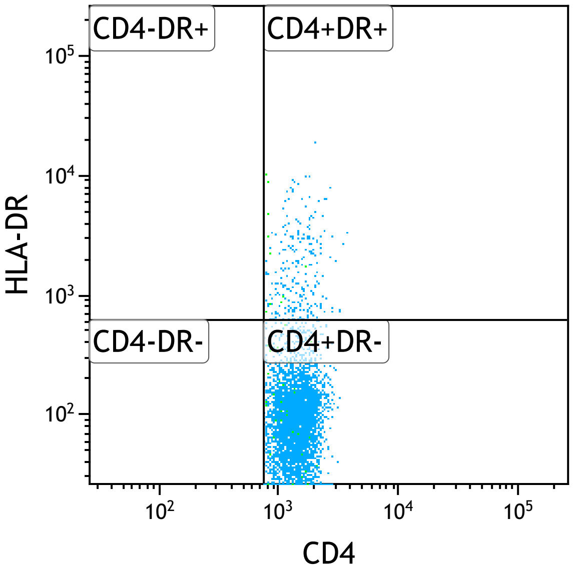

[CD3+CD8+] CD8 / CD28

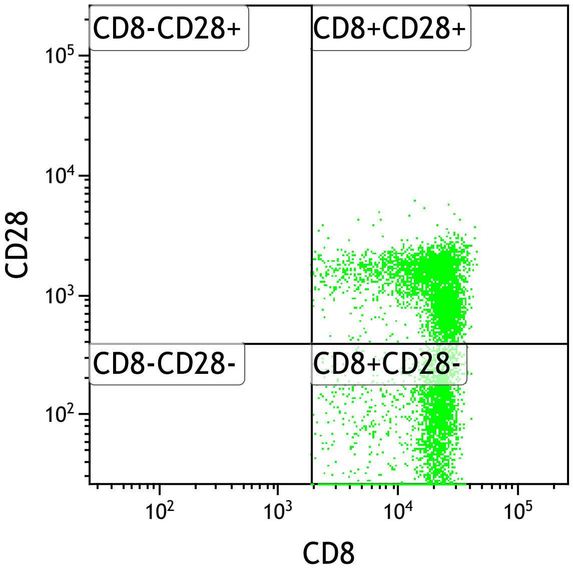

[CD3+CD8+] CD8 / CD38

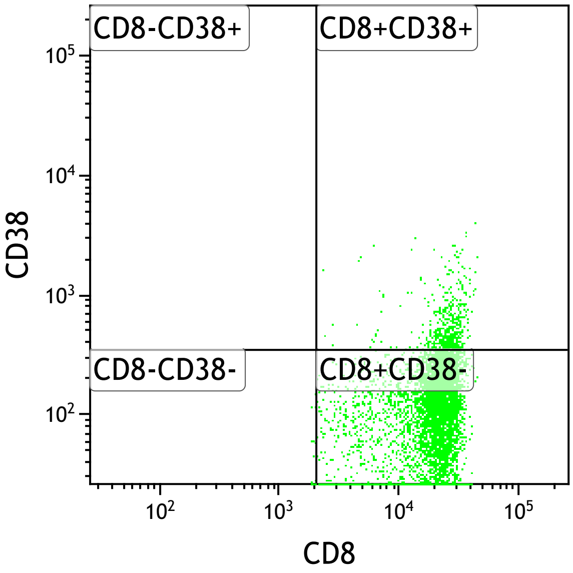

[CD3+CD8+] CD8 / HLA-DR

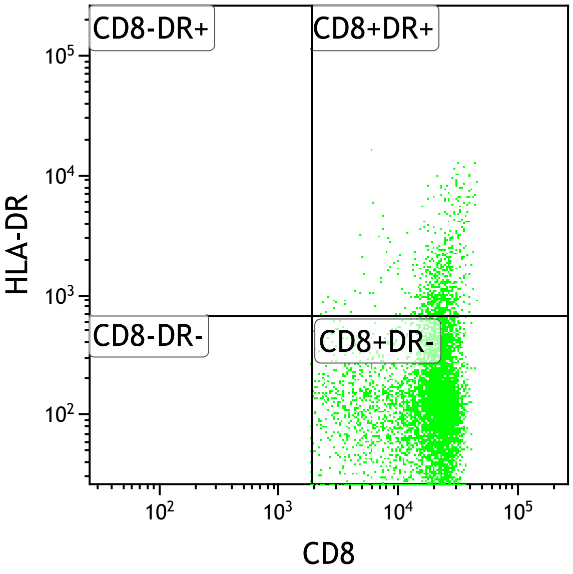

Gate	Number	%Total	%Gated	Logic
All	60,102	100.00	100.00	Ungated
H	10,014	16.66	16.66	H
LYM	28,708	47.77	47.77	LYM
CD3+CD4-	9,239	15.37	32.18	CD3+CD4- AND LYM
CD3+CD4+	5,892	9.80	20.52	CD3+CD4+ AND LYM
CD4+CD28-	212	0.35	3.60	CD4+CD28- AND CD3+CD4+ AND LYM
CD4+CD28+	5,680	9.45	96.40	CD4+CD28+ AND CD3+CD4+ AND LYM
CD4+CD38-	4,485	7.46	76.12	CD4+CD38- AND CD3+CD4+ AND LYM
CD4+CD38+	1,407	2.34	23.88	CD4+CD38+ AND CD3+CD4+ AND LYM
CD4+DR-	5,637	9.38	95.67	CD4+DR- AND CD3+CD4+ AND LYM

[Ungated] CD45 / SSC-A

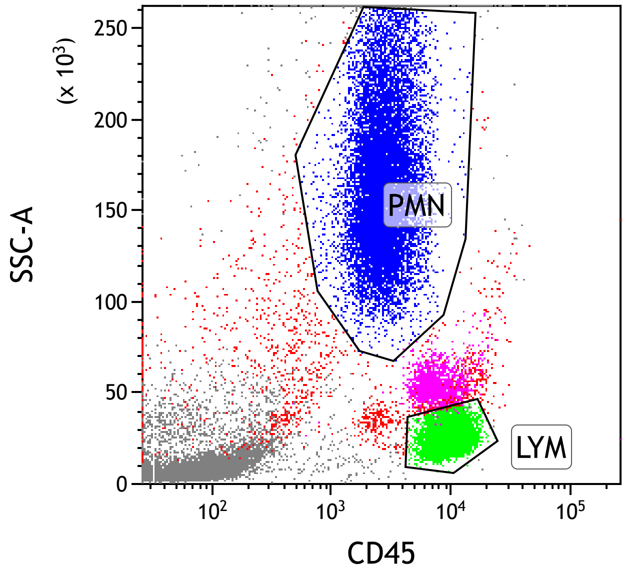

[Ungated] CD14 / SSC-A

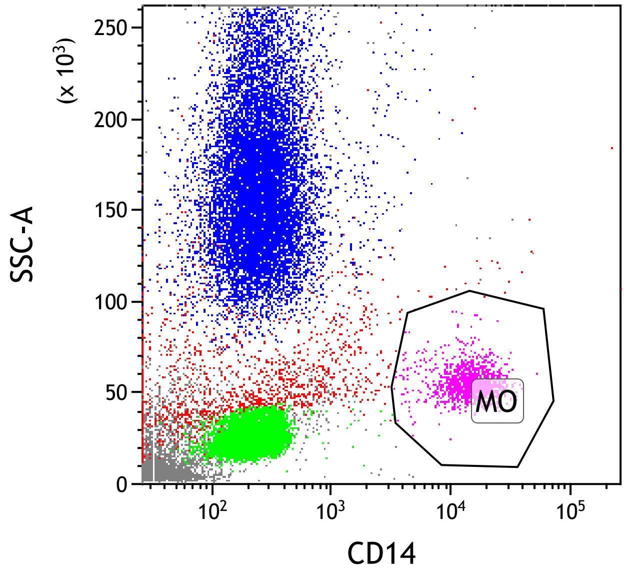

[Ungated] FSC-A / SSC-A

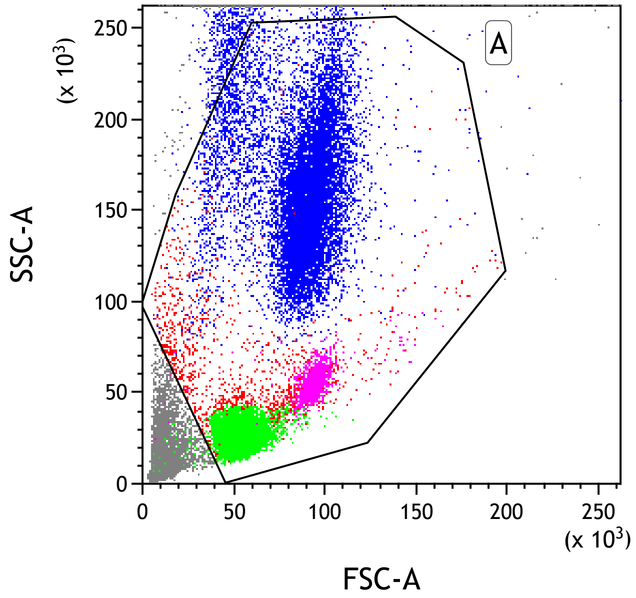

[A] CD64

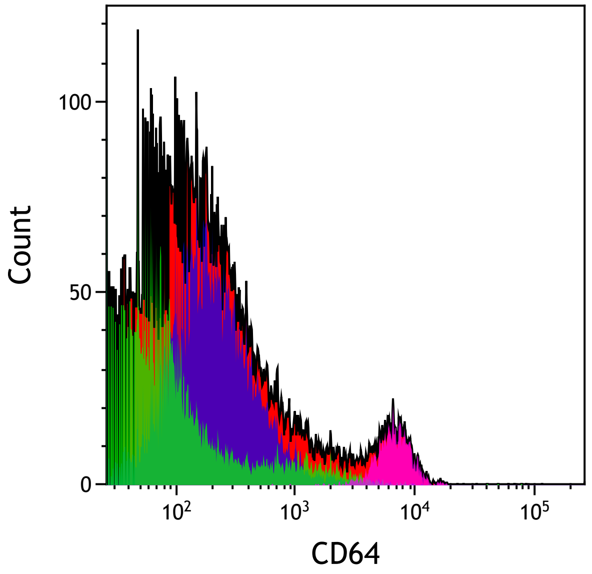

Gate	Number	%Gated	X-Med
All	29,060	100.00	112.01

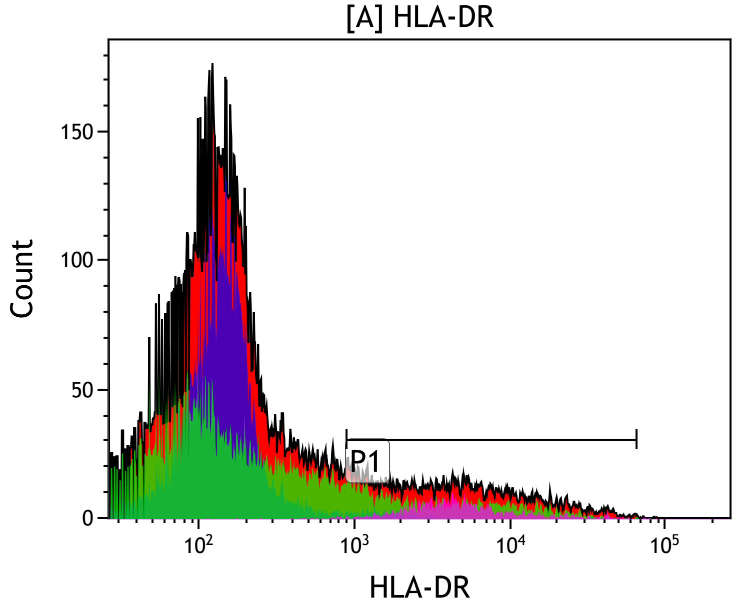

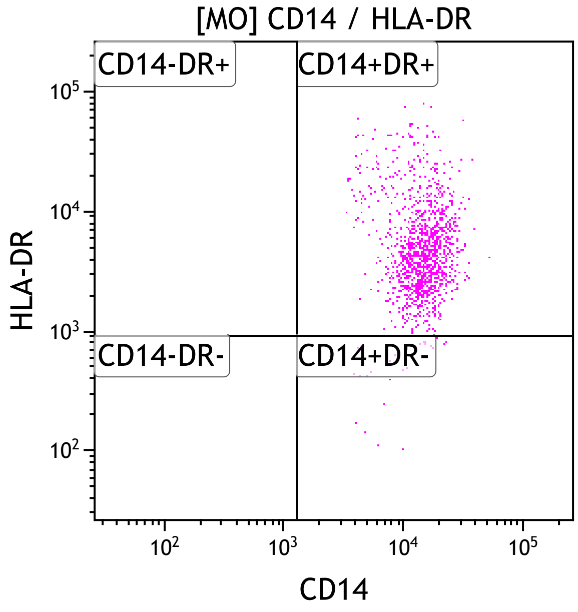

Gate	Number	%Total	%Gated	Logic
All	50,000	100.00	100.00	Ungated
A	29,060	58.12	58.12	A
P1	4,641	9.28	15.97	P1 AND A
LYM	13,820	27.64	27.64	LYM
MO	1,290	2.58	2.58	MO
CD14+DR-	33	0.07	2.56	CD14+DR- AND MO
CD14+DR+	1,257	2.51	97.44	CD14+DR+ AND MO
CD14-DR-	0	0.00	0.00	CD14-DR- AND MO
CD14-DR+	0	0.00	0.00	CD14-DR+ AND MO
PMN	12,871	25.74	25.74	PMN

[Ungated] CD45 / CD64

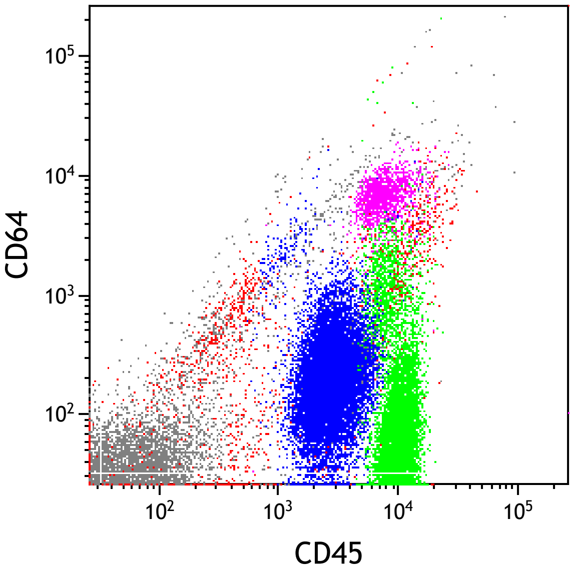

20220218ICU_11-1ICU
Protocol Treg - Plot Sheet 1

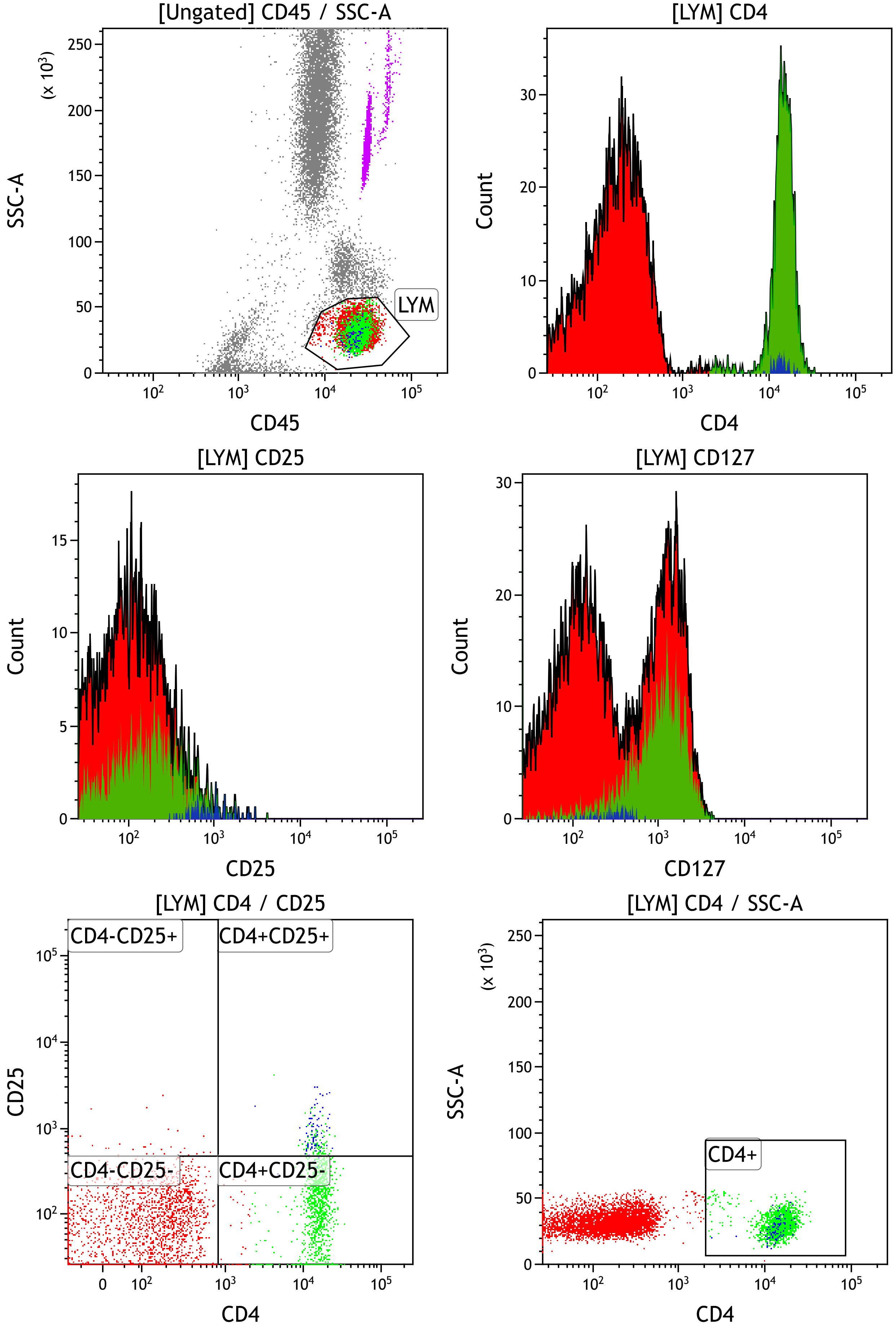

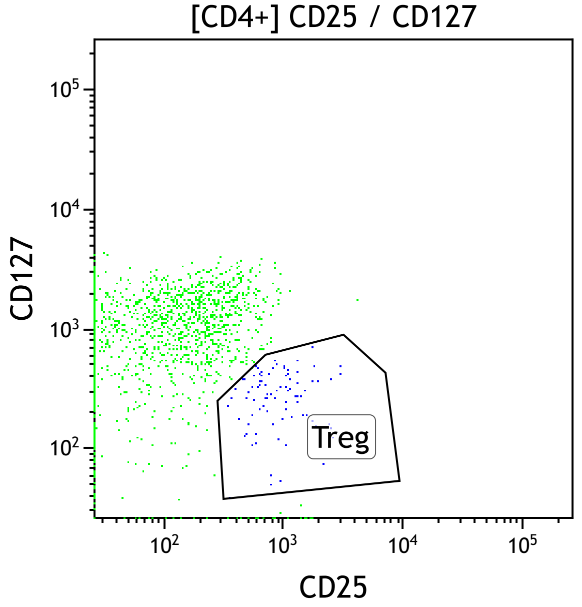

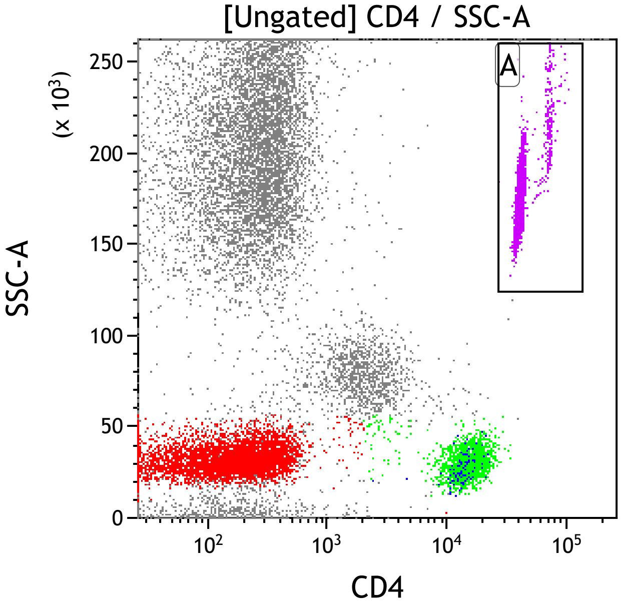

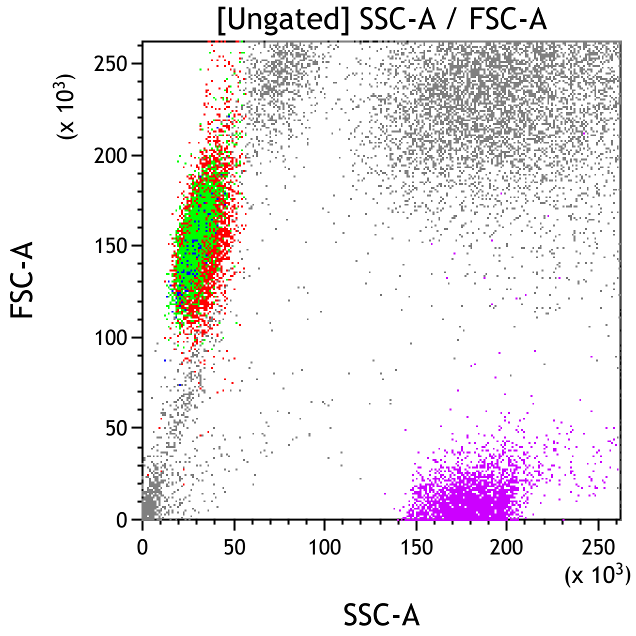

Gate	Number	%Total	%Gated	Logic
All	25,545	100.00	100.00	Ungated
A	3,494	13.68	13.68	A
LYM	9,670	37.85	37.85	LYM
CD4+	2,107	8.25	21.79	CD4+ AND LYM
Treg	90	0.35	4.27	Treg AND CD4+ AND LYM
CD4+CD25-	1,978	7.74	20.46	CD4+CD25- AND LYM
CD4+CD25+	171	0.67	1.77	CD4+CD25+ AND LYM
CD4-CD25-	7,486	29.31	77.41	CD4-CD25- AND LYM
CD4-CD25+	35	0.14	0.36	CD4-CD25+ AND LYM
